# Tracheal Sounds Acquisition Using Smartphones

**DOI:** 10.3390/s140813830

**Published:** 2014-07-30

**Authors:** Bersain A. Reyes, Natasa Reljin, Ki H. Chon

**Affiliations:** Department of Biomedical Engineering, Worcester Polytechnic Institute, 100 Institute Road, Worcester, MA 01609, USA; E-Mails: bareyes@wpi.edu (B.A.R.); nreljin@wpi.edu (N.R.)

**Keywords:** respiratory sounds, tracheal sounds, smartphone, respiratory rate, breath-phase, entropy, time-frequency representation

## Abstract

Tracheal sounds have received a lot of attention for estimating ventilation parameters in a non-invasive way. The aim of this work was to examine the feasibility of extracting accurate airflow, and automating the detection of breath-phase onset and respiratory rates all directly from tracheal sounds acquired from an acoustic microphone connected to a smartphone. We employed the Samsung Galaxy S4 and iPhone 4s smartphones to acquire tracheal sounds from *N* = 9 healthy volunteers at airflows ranging from 0.5 to 2.5 L/s. We found that the amplitude of the smartphone-acquired sounds was highly correlated with the airflow from a spirometer, and similar to previously-published studies, we found that the increasing tracheal sounds' amplitude as flow increases follows a power law relationship. Acquired tracheal sounds were used for breath-phase onset detection and their onsets differed by only 52 ± 51 ms (mean ± SD) for Galaxy S4, and 51 ± 48 ms for iPhone 4s, when compared to those detected from the reference signal via the spirometer. Moreover, it was found that accurate respiratory rates (RR) can be obtained from tracheal sounds. The correlation index, bias and limits of agreement were *r^2^* = 0.9693, 0.11 (−1.41 to 1.63) breaths-per-minute (bpm) for Galaxy S4, and *r^2^* = 0.9672, 0.097 (–1.38 to 1.57) bpm for iPhone 4s, when compared to RR estimated from spirometry. Both smartphone devices performed similarly, as no statistically-significant differences were found.

## 1. Introduction

Respiratory sounds vary and they include breath sounds, adventitious sounds, and sounds from the respiratory muscles, excluding voiced sounds during breathing, according to the European Respiratory Society (ERS) Task Force Report [1]. Lung sounds are all respiratory sounds heard or detected over the chest wall or within the chest, including breathing and adventitious sounds detected at this location [1]. Tracheal sounds are those heard or detected over the extrathoracic part of the trachea [1]. In this study, we will concentrate only on the study of tracheal sounds recorded from healthy subjects.

Tracheal sounds exhibit characteristics of noise dynamics with a broad-band spectrum and contain several resonance peaks [2]. Tracheal sounds exhibit well defined inspiratory and expiratory phases and their frequency contents are higher compared to lung sounds [3]. It has been found that inspiratory and expiratory phases have similar frequency contents for tracheal sounds [3,4]. Turbulent flow in upper airways is primarily responsible for generation of tracheal sounds, thus, their characteristics are influenced by airway dimensions [5]. It has been shown that tracheal sounds consist of a dominating local turbulent eddy and a propagating acoustic component with resonances [2]. The relation between airflow, *F*, and tracheal sound's amplitude, *A*, has been recognized. A power law of the form *A* = *kF^α^* is considered the typical best fit, where *k* and *α* are constants with varying values having been found from different research groups [2,6–9].

The stethoscope remains the most widely used instrument in clinical medicine and its use during auscultation still guides in diagnosis when other pulmonary function testing is not available [10]. However, auscultation with the mechanical stethoscope has limitations [3,11,12]. Namely, it is a subjective process that depends on the skill of the physician [13]; it is limited by the human auditory system [14]; it depends on the stethoscope model used, and the stethoscope itself is more adequate for cardiac auscultation [3]; and the respiratory sounds are not permanently recorded for further analysis.

Over the last decades, some limitations of the stethoscope have been overcome by using Computerized Respiratory Sound Analysis (CORSA). Computerized analysis of respiratory sounds has led to the renaissance of lung auscultation over the last decades but this renewed interest has also produced several different measurement systems by different laboratories [5,15,16]. Fortunately, standardization of CORSA has been addressed, and guidelines for the minimum requirements of CORSA systems have been provided [17,18]. The European Community financed the CORSA project, which explicitly expressed that [19] “one goal of the current technological developments is to combine processing power, storage, miniaturization of components and analysis programed into a small hand-held computerized stethoscope that will provide the clinician with much more useful information than the current simple mechanical stethoscope.” Given the need for reliable devices that can record and analyze respiratory sounds in a continuous, non-invasive, and portable fashion, we propose to develop a respiratory sound system based on a smartphone platform.

It is well known that the use of smartphones has become popular and that they are widely available and used for everyday activities including vital sign measurements. By taking advantage of the smartphone's processing power, peripheral noninvasive and cost-effective sensors, and wireless communications capabilities, recent efforts have been made to create various medical applications for self-monitoring. In particular, our research group has made efforts to employ smartphones for health monitoring in the area of cardiac monitoring [20,21].

The development of an inexpensive, reliable, and portable CORSA system would expand the noninvasive diagnostic capabilities of the auscultation procedure when used by general practitioners and pneumologists in the diagnosis of respiratory diseases during the clinical examination. The use of an inexpensive, reliable, and portable system would also enable more health centers to undertake the quantitative analysis of respiratory sounds for diagnosis of respiratory diseases. We hypothesize that a CORSA system that satisfies these characteristics can be implemented using a smartphone.

There have been attempts to develop a portable system for the analysis of respiratory sounds. In particular, the concept of a portable device based on a microcontroller, memory arrays, and liquid crystal displays has been proposed but without sufficient details about implementation results [22]. The concept of a digital stethoscope using a palmtop computer has been also proposed but neither technical detail about the characteristics of the system that guarantee the reliability of the acquired respiratory sound signals nor examples of the acquired signals were provided [23]. Recently, the concept of a smartphone-based asthma monitoring system has been proposed [24,25]. The sounds were processed via custom-designed hardware and the obtained information was wirelessly transmitted to the smartphone to display the processed data. The processed data were then transmitted to a medical database via the Internet [24]. Sounds obtained from Internet sources were transmitted to smartphone and reconstruction techniques were tested [25]. However, like in the previous attempts, no information was provided about the reliability of the system when acquiring real respiratory sounds.

The smartphone-based CORSA system we propose differs from the existing systems in two main ways. First, the signal processing of the acquired respiratory sounds will be performed directly on the smartphone, without the employment of complicated secondary devices with microcontroller-based architectures that increase the energy consumption and the cost of maintenance/upgrade. The smartphone will be used not only to display the respiratory sound signal, but will also control the acquisition stage and perform the signal processing. Second, no wireless communication will be used to transmit the acquired respiratory sounds to the smartphone in order to avoid losses in the quality of the transmitted information and to reduce the energy consumption in the preprocessing stage. The proposed mobile system will be designed to satisfy the standard requirements for a CORSA system and will take advantage of the already available hardware characteristics of the smartphone for the acquisition, visualization, and processing of the respiratory sounds. The proposed system will have the advantage of being non-invasive, low cost, and a portable device which can be used to monitor anytime and anywhere. It should be noted that the developed system was only used to acquire tracheal sounds while the raw recordings were transferred and processed on a computer.

In this paper, the reliability of our proposed smartphone-based system will be tested on the well-known characteristics of the tracheal sounds: well-defined breath phases, similar frequency content for the inspiratory and expiratory phases, and a flow-dependent amplitude relationship. In addition, we aim to detect the breath-phase onsets from the smartphone-acquired tracheal sounds and compare the results with those obtained using the flow signal from a spirometer which is considered the reference. Finally, we will estimate respiration rates from the smartphone-acquired tracheal sound signals and validate them using the respiration rates estimated from the volume changes derived from a spirometer.

## 2. Material and Methods

### 2.1. Subjects

Nine healthy non-smoker volunteers (seven males and two females) ages ranging from 23 to 35 years (mean ± standard deviation: 27.9 ± 5.1), weight 68.7 ± 8.1 kg and height 170.7 ± 6.7 cm, were recruited for this study. The study group consisted of students and staff members from Worcester Polytechnic Institute, MA, USA. All volunteers were invited to participate in the study and each consented to be a subject and signed the study protocol approved by the Institutional Review Board of WPI.

### 2.2. Tracheal Sounds Data Acquisition

#### Equipment

Tracheal sounds were acquired using an acoustical sensor composed of a subminiature electret microphone BT-21759-000 (Knowles Electronics, Itasca, IL, USA) encased in a plastic bell. This microphone operates with a voltage supply ranging from 1.3 to 10 V with a low amplifier current drain of 50 μA, provides a flat frequency response between 50 and 3000 Hz, and offers advantages in terms of high durability compared to contact sensors. A light plastic bell was used for air-coupling between the sensor and the recording area on the surface over the trachea. The plastic bell consisted of a conical coupler chamber. This shape provides an efficient transducer of air pressure fluctuations from the skin over the trachea to the microphone [3]. This acoustic sensor was developed by our colleagues at the Metropolitan Autonomous University at Mexico City, Mexico, and had been successfully used for respiratory sound acquisition applications [26,27]. Acoustical sensors of similar characteristics have been found to be adequate for respiratory sound research [17,28,29]. To minimize power line electrical interference, shielded twisted pair cables were used to connect the acoustical sensor to the standard 3.5 mm audio jack in the smartphone. In order to provide impedance matching and to obtain a balance between saturation and sensitivity, a simple voltage divider composed of two resistors of 2.2 kΩ was used before transmitting the recorded tracheal sounds to the smartphone. We cabled to the standard 3.5 mm audio connector to avoid high power consumption or loss of quality due to wireless communication.

Two smartphones were selected for this research: (1) the Galaxy S4 manufactured by Samsung (Samsung Electronics Co., Seoul, South Korea) and running an Android v4.4.2 operating system, and (2) the iPhone 4s manufactured by Apple (Apple, Inc., Cupertino, CA, USA) and running an iOS 6.1 operating system. Selection of the devices was made based on the high market share of each phone's product family, and the dominant combined market share of their operating systems. In addition, each device contains a high-fidelity audio system that satisfies the minimum requirements recommended by the ERS Task Force Report [18]. The tracheal sounds were recorded using the corresponding built-in audio recorder application of each smartphone (Voice Recorder in the Galaxy S4, and Voice Memos in the iPhone 4s) using the predetermined 16-bit per sample and 44.1 kHz sampling rate and saved in the native .m4a format in each device. Recorded audio files were transferred to a personal computer and converted to .wav format preserving the same bits per sample and sampling rate using a conversion software (Free Audio Converter v.5.0.33, DVDVideoSoft Ltd., United Kingdom) and stored for further processing in Matlab (R2012a, The Mathworks, Inc., Natick, MA, USA).

Simultaneously with the tracheal sounds, the airflow was recorded using a spirometer system consisting of a respiratory flow head (MLT1000L, ADInstruments, Inc., Dunedin, New Zealand) connected to a differential pressure transducer to measure airflow (FE141 Spirometer, ADInstruments, Inc., Dunedin, New Zealand). The airflow signal was digitized using a 16-bit A/D converter PowerLab/4SP, ADInstruments, Inc., Dunedin, New Zealand) at 10 kHz sampling rate by using the manufacturer's software (LabChart 7, ADInstruments, Inc., Dunedin, New Zealand). The volume signal was computed online as the integral of the airflow. Prior to each day of recordings, the spirometer system was calibrated using a 3 L calibration syringe (Hans Rudolph, Inc., Shawnee, KS, USA). A new set of disposable filter, reusable mouthpiece, and disposable nose clip (MLA304, MLA1026, MLA1008, ADInstruments, Inc., Dunedin, New Zealand) was given to each subject.

#### Acquisition protocol

**Figure 1.**
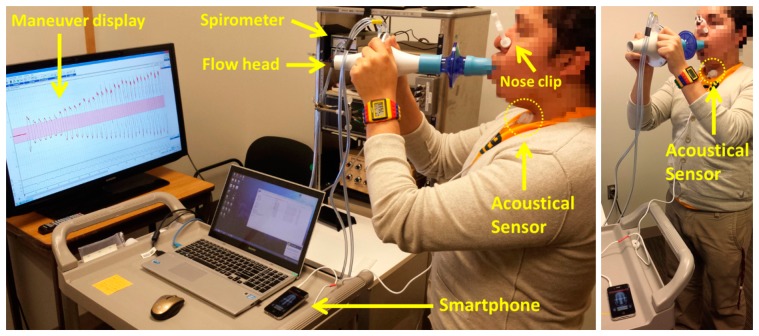
Tracheal sound recording using a smartphone and simultaneous recording of the airflow signal via spirometer during the respiratory maneuver. The acoustical sensor transmitted the tracheal sound to the smartphone via the standard 3.5 mm audio connector.

Experiments were performed not in an anechoic chamber but in an office room held quiet. The acoustical sensor was fixed to the neck of the volunteers at the anterior cervical triangle using a double-sided adhesive ring (BIOPAC Systems, Goleta, CA, USA) to avoid pressure variations if hand-placed. Subjects were asked to breathe through a spirometer for approximately 2 min at airflow levels above 0.5 L/s and at maximum of around 2.5 to 3.0 L/s; varying among subjects. The subjects were instructed to breathe first by increasing volumetric flow rates with each breath, and then with decreasing volumetric flow rates with each breath. The airflow was displayed on a 40″ monitor placed in front of the subject in order to provide visual feedback. Visual markers were placed between −0.5 to 0.5 L/s and the subjects were instructed to keep the airflow peaks of each respiratory phase outside this boundary area. Initial inspiratory and final expiratory apnea phases of approximately 5 s were acquired in order to record the ambient noise levels. Nose clips were used to clamp the nostrils during the respiratory maneuver. An example of the acquisition protocol is shown in Figure 1. A respiratory maneuver was acquired using each smartphone in a sequential way, where the order of the devices was randomized between subjects.

### 2.3. Data Pre-Processing

The acquired tracheal sounds were initially down-sampled from 44.1 kHz to 6300 Hz as this frequency still satisfies the Nyquist criteria and reduces the computational burden. Then, the tracheal sounds were digitally filtered using a 4th-order Butterworth filter with a passband between 100 to 3000 Hz to minimize the heart sounds and muscle interferences. The filter was applied in forward and backward scheme to produce zero-phase distortion and minimize the start and end transients. The airflow and the volume signal were down-sampled to 5 kHz and then interpolated to achieve the same sampling frequency of 6300 Hz, and finally they were lowpass filtered at 20 Hz to minimize high frequency components due the interpolation processes that are not related to the respiratory maneuver.

**Figure 2.**
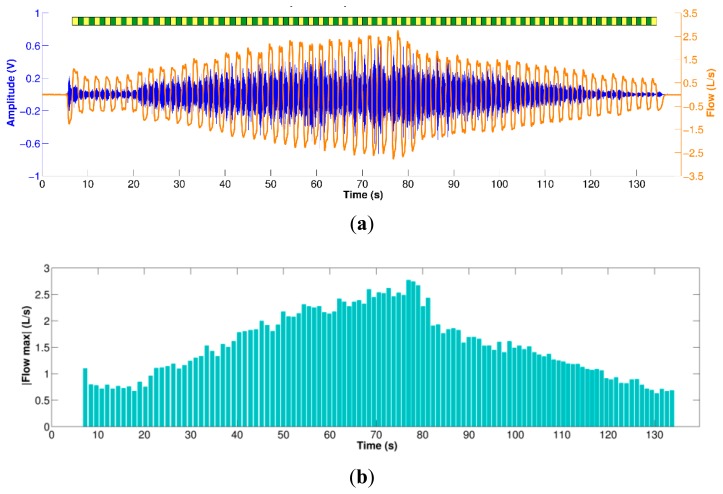
Tracheal sounds acquired using a smartphone during the respiratory maneuver. (**a**) Preprocessed tracheal sound (in Volts) aligned with the corresponding spirometer's airflow signal. Yellow and green bars on top indicate the inspiratory and expiratory phases, respectively; (**b**) Corresponding absolute airflow peaks for each respiratory phase of the maneuver.

The volume signal was used for automatic segmentation of the inspiratory and expiratory phases by finding its corresponding local maxima and minima during the respiratory maneuver (breath-phase onsets) and by computing the volume slope between both consecutive onsets (positive for inspiration and negative for expiration). Although both the tracheal sounds and the volumetric flow rate were simultaneously recorded, due to different time delays and press start button times of the smartphone, these signals were manually aligned and their durations corrected to the minimum length of both. An example of the filtered, aligned and segmented tracheal sounds and airflow measured via spirometer is shown in Figure 2 for the respiratory maneuver performed by one subject.

### 2.4. Tracheal Sound Amplitude and Airflow Relationship

Although visual inspection of the acquired tracheal sounds indicates that their amplitude increases as airflow increases, and decreases as airflow decreases, we used the information from automatically extracted inspiratory and expiratory phases to quantify this relationship. At each respiratory phase, the peak airflow was found and a time window of 400 ms was created starting at the time instant when the airflow signal reached the upper 10% of the airflow, where it reached its plateau. The tracheal sound segments within these windows were extracted and their corresponding power spectral density (PSD) was computed using the fast Fourier transform (FFT) with *NFFT* = 1024. The PSD of the initial apnea period was also computed and subtracted from each PSD of the tracheal sounds segment. The area under the curve of the resulting PSD was computed and regarded as the amplitude of the tracheal sound for that corresponding respiratory breath-phase. For each subject, the inspiratory/expiratory amplitudes were normalized by dividing them by the average inspiration/expiration amplitude [2]. Finally, for each subject, the best fitting curve of the form *A* = *kF^α^* was computed separately for the inspiratory and expiratory phases.

### 2.5. Breath-Phase Onset Detection Using Tracheal Sounds

Tracheal sounds acquired with the smartphones were used to estimate the breath-phase onset via the Shannon entropy approach. The Shannon entropy of tracheal sounds has been used as a method for estimating the airflow [30,31]. The Shannon entropy (SE) of a random signal with probability density function (pdf) *p* is defined as

(1) $$SE\left(p\right)=-\sum_{i=1}^{N}p_{i}\cdot \text{log}(p_{i})$$

where *N* is the number of outcomes of the random variable with pdf *p*. The SE is used to quantify the uncertainty or irregularity of the process [32]. It has been found that the entropy values quantify the standard deviation and correlation properties of the signal where the individual weight contributions are not trivial to separate [33,34].

As proposed for the airflow estimation from tracheal sounds, we applied the Shannon entropy in a moving window scheme as follows. First, the recorded tracheal sounds were sequestered into 25 ms windows with 50% overlap between successive windows. For each of the resulting windows, the Shannon entropy was computed. One way to estimate the pdf *p* is to use the histogram. However, due to the low number of samples within each overlapping window (*n* = 157 samples) its accuracy would be low. Instead, the pdf of each windowed tracheal sound was computed using the Parzen-window density estimation method with a Gaussian kernel [35,36]. This non-parametric method estimates the pdf *p* of the random sample *x* from which the sample was derived, by superposing window functions placed at each of *n* observations and determining how many observations *x_i_* fall within the specified window *h*, *i.e*., the contribution of each observation *x_i_* within this window *h*. Then, the pdf is estimated as the sum of the total of the contributions from the observations to this window, and the Parzen-window estimate *p̂* is given by

(2) $$\hat{p}(x)=\frac{1}{n}\sum_{i=1}^{n}\frac{1}{h}K\left(\frac{x-x_{i}}{h}\right)$$

where *h*>0 is the window width of the kernel *K*, which is typically a pdf itself. When a Gaussian kernel is used, the Parzen-window estimate becomes

(3) $$\hat{p}(x)=\frac{1}{n}\sum_{i=1}^{n}\frac{1}{h\sqrt{2\pi }}\text{exp}\left(-\frac{1}{2}{\left(\frac{x-x_{i}}{h}\right)}^{2}\right)$$

where *h* is the standard deviation of the Gaussian kernel, and was set to [37]

(4) $$h=1.06\cdot SD(x)\cdot n^{-\frac{1}{5}}$$

with *SD*(·) being the standard deviation of the windowed tracheal sound.

**Figure 3.**
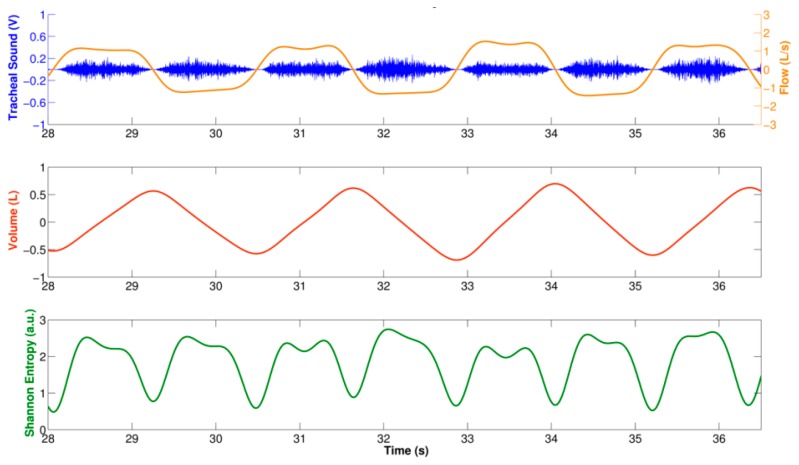
Shannon Entropy of the tracheal sounds acquired using a smartphone. **Top**: Segment of tracheal sound and corresponding airflow from spirometer (positive lobes are inspirations and negative lobes are expirations); **Middle**: Volume signal obtained with the spirometer as the integral of the flow; **Bottom**: Shannon entropy of tracheal sound. Observe that local minima of the Shannon entropy are obtained around the onset of each respiratory phase.

The SE estimated from each windowed tracheal sound was assigned to the middle time point of the window, and was interpolated using cubic spline in order to recover the original duration of the tracheal and volumetric flow rate signals. Figure 3 shows an example of the computed SE using the described approach for a tracheal sound segment acquired using a smartphone. Observe that this SE signal from a smartphone resembles a rectified airflow signal, as has previously been found when the SE of tracheal sounds are used for airflow estimation purposes [30,31]. In order to estimate the breath-phase onset, the SE signal was inverted and the corresponding local maxima were automatically detected. First, the SE signal was normalized between [0–1] and down-sampled to 7.875 Hz. The PSD of the down-sampled SE signal was computed with Welch's modified periodogram method with a Hamming window, with 50% overlap, and *NFFT* = 512 bins. The peak of the PSD and its corresponding frequency *f_peak_* were found. The local maxima of the SE signal were found and all those maxima that did not satisfy the threshold values criteria were removed. The amplitude threshold was set to *thr*_1_ = 0.1, and the time threshold was set to *thr*_2_ = 0.5*1/*f_peak_*. Finally, the corresponding time onsets computed from the down-sampled SE were mapped to the closest point of the original SE time series which had a time resolution of Δ*_t_*≈0.159*ms* given the sampling frequency *f_s_* = 6300*Hz*. The detected breath-phase onsets from tracheal sounds acquired from each smartphone were compared to those computed from volume.

### 2.6. Instantaneous Respiratory Rate Estimation Using Tracheal Sounds

As previously stated, the SE of the tracheal sounds resembles the rectified airflow signal. This SE has two lobes for each breathing cycle indicated by the volume signal; see Figure 3. We took advantage of this fact to estimate the instantaneous respiratory rate from tracheal sounds acquired with the smartphone. In particular, we employed a joint time-frequency representation (TFR) approach. In general, a TFR allows one to analyze which frequencies of a signal under study are present at a certain time, *i.e*., a TFR describes the energy density of a signal simultaneously in the time and frequency domains [38]. This characteristic is useful when analyzing signals whose frequency content varies with time, as is the case for the respiratory rate.

The most widely-used TFR in the respiratory sounds field is the spectrogram (SP) given by the magnitude square of the short time Fourier transform (STFT) [4,10]. The idea behind the SP is that in order to study the properties of the signal *s* around time *t*, the original signal around that time is emphasized but it is suppressed at other times by multiplying by a window function *w*(*t*) centered at *t*, to produce a modified signal *s_t_(τ)* given by [38,39]

(5) $$s_{t}(\tau )=s(\tau )w(\tau -t)$$

where the modified signal is a function of two times, the fixed time *t* of interest, and the time *τ*. The window function allows the modified signal to satisfy

(6) $$\begin{array}{cc} s_{t}\left(\tau \right)= & \{\begin{array}{cc} s\left(\tau \right) & \text{for}\;\tau \;\text{close to}\;t\\ 0 & \text{for}\;\tau \;\text{far away from}\;t \end{array} \end{array}$$

Given that the modified signal emphasizes the original signal around time *t*, its Fourier transform reflects the frequency distribution around that time

(7) $$\begin{array}{l} S_{t}(\omega )=\frac{1}{\sqrt{2\pi }}\int e^{-j\omega \tau }s_{t}(\tau )d\tau \\ S_{t}(\omega )=\frac{1}{\sqrt{2\pi }}\int e^{-j\omega \tau }s(\tau )w(\tau -t)d\tau \end{array}$$

and hence the name of STFT. The corresponding spectral density at time *t* is given by

(8) $$SP(t,\omega )=|S_{t}(\omega ){|}^{2}={\left|\frac{1}{\sqrt{2\pi }}\int e^{-j\omega \tau }s(\tau )w(\tau -t)d\tau \right|}^{2}$$

where a spectrum is obtained at each time instant and the total of that spectrum is the time-frequency distribution of the original signal *SP*(*t,ω*). This distribution has received different names depending on the application field, *e.g.*, respirosonogram in the respiratory sounds field [10], but the most common is simply spectrogram.

The SP was applied to the volume signal and to the SE of the acquired tracheal sounds. Due to the very low frequency content of the respiratory rate compared to the original sampling frequency, both signals were down-sampled to 7.875 Hz. The SP was computed using *NFFT* = 512 frequency bins, and a Hamming window of 10 s duration. The resulting TFR was normalized between [0–1] and at each time instant, the maximum peak was computed around the central frequency of the whole signal and the corresponding frequency vector was extracted. Due to the discussion mentioned above, the frequency vector extracted from the volume was regarded as the reference instantaneous respiratory frequency, while the half of the frequency vector extracted from the SE signal corresponded to the instantaneous frequency estimated from each smartphone. All instantaneous respiratory frequencies were converted from Hz to breaths-per-minute (bpm).

For each smartphone, three performance indices were computed for the instantaneous respiratory rate (IRR) of each subject. The first index corresponds to the cross-correlation coefficient *ρ* between the IRR obtained with the corresponding smartphone and the one obtained from the volume from spirometer given by

(9) $$\rho =\frac{\sum_{i=1}^{N}{\text{IRR}}_{\text{volume}}(i)\cdot {\text{IRR}}_{\text{smartphone}}(i)}{\sqrt{\sum_{i=1}^{N}{\left({\text{IRR}}_{\text{volume}}(i)\right)}^{2}\cdot \sum_{i=1}^{N}{\left({\text{IRR}}_{\text{smartphone}}(i)\right)}^{2}}}$$

where *IRR_volume_* represents the instantaneous respiratory rate obtained from the volume, *IRR_smartphone_* the corresponding IRR estimated from the tracheal sound acquired with the iPhone 4s or Galaxy S4 smartphone, and N is the length of the time vector of the signal. Observe that if the *IRR_volume_* and *IRR_smartphone_* are the same, the value of *ρ* is unitary. Therefore, *ρ* values close to 1 reflect a good estimation performance. The remaining two indices computed were the root-mean-squared error *RMSE*, and the normalized root-mean-squared error *NRMSE*, given by

(10) $$\text{RMSE}=\sqrt{\frac{{\left(\sum_{i=1}^{N}{\text{IRR}}_{\text{volume}}(i)-{\text{IRR}}_{\text{smartphone}}(i)\right)}^{2}}{N}}$$

(11) $$\text{NRMSE}=\frac{\text{RMSE}}{\text{mean}({\text{IRR}}_{\text{volume}})}\times 100\%$$

respectively.

## 3. Results and Discussion

**Figure 4.**
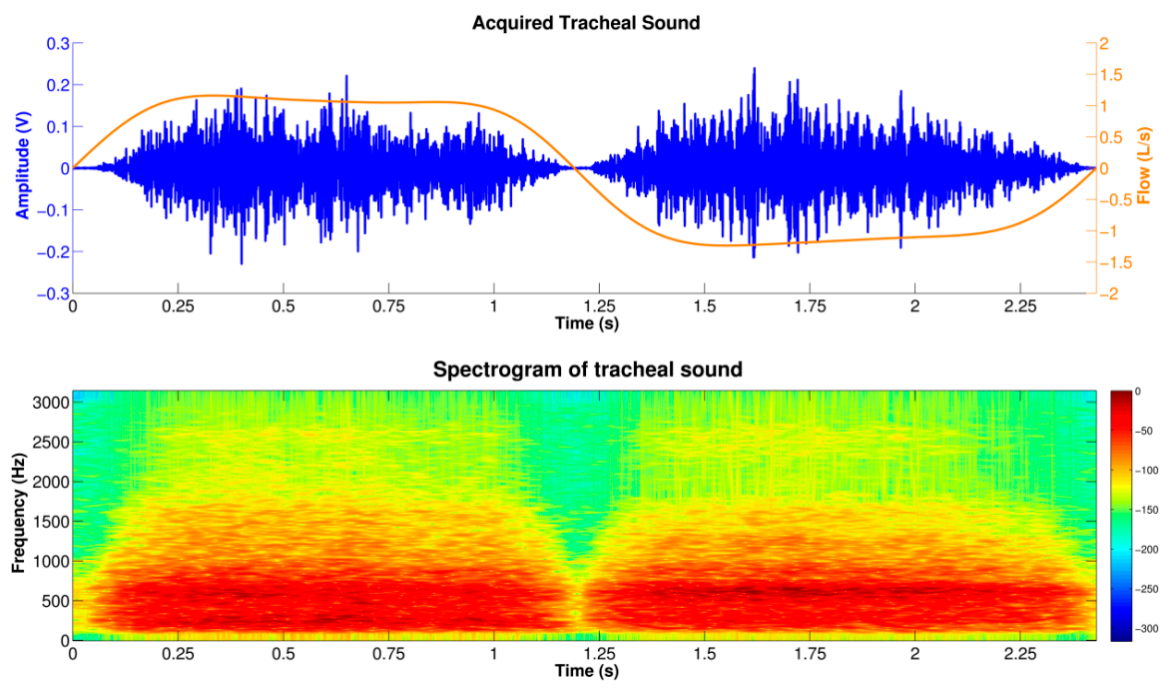
Time-frequency characteristics of the tracheal sounds acquired using the smartphone during one respiratory cycle. **Top**: Tracheal sound waveform together with its corresponding airflow signal (positive and negative lobes indicate the inspiration and expiration, respectively); **Bottom**: Time-frequency representation of the acquired tracheal sound computed via the spectrogram using a 100 ms Hamming window. Red/blue color in the color map indicates high/low intensity in decibels.

The tracheal sound signals acquired using both the Galaxy S4 and the iPhone 4s showed a temporal intensity variation related to the airflow during the respiratory phases as shown in Figure 4. The TFR of the smartphone-acquired tracheal sounds (bottom panel of Figure 4) shows characteristics of broad band noise where both inspiratory and expiratory phases have their main frequency components not higher than 1.5 kHz, with a sharp drop in power around 800 Hz, which is in agreement with other studies [40]. In addition, both respiratory phases have similar frequency content for similar airflow peaks and a silent period separating both phases could also be observed from both the tracheal sound waveform as well as its TFR. These results are in agreement with the findings reported in the literature when using CORSA systems [3,4,10].

In the next subsections we present the results obtained for both smartphones for the tracheal sound's amplitude with airflow, the breath-phase onset detection, and the respiratory rate estimation.

### 3.1. Tracheal Sound Amplitude and Airflow Relationship

**Figure 5.**
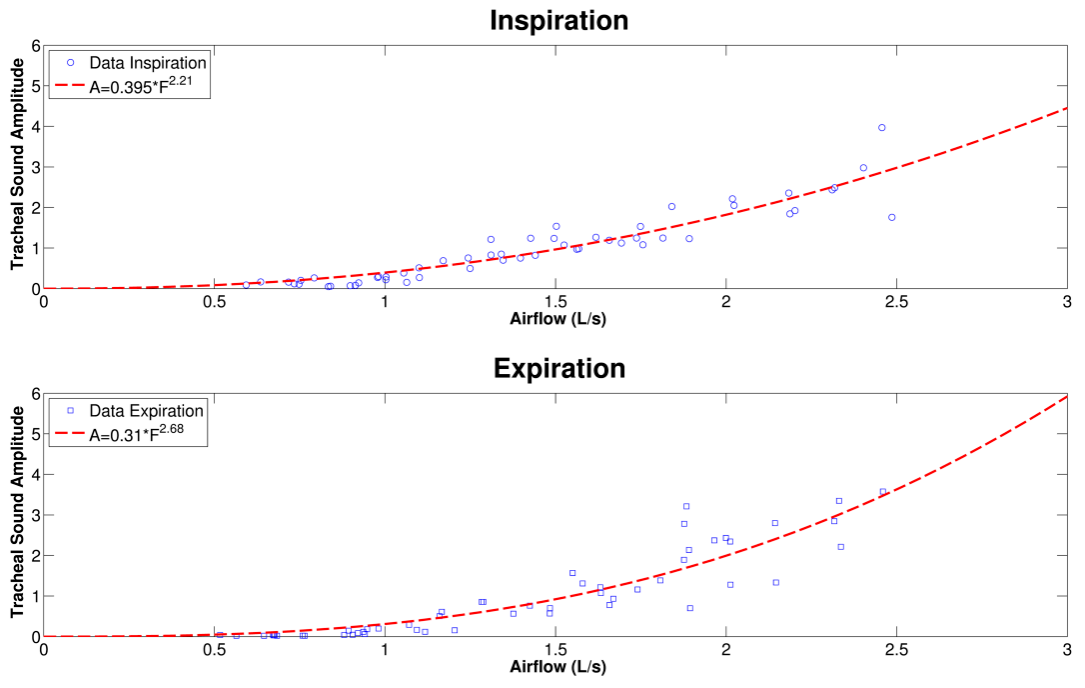
Example of smartphone-acquired tracheal sounds amplitude as a function of airflow during the inspiratory and expiratory phases for one subject. Red dashed lines correspond to the best fit curves of the form *A* = *kF^α^*.

A representative example of the curve fitting of the tracheal sound *versus* airflow acquired with an iPhone 4s and spirometer, respectively, for the inspiration and expiration phases is shown in Figure 5. We observe that during the inspiratory and expiratory phases, the increasing tracheal sounds' amplitudes as flow increases follow a power law relationship. The results of the power law model fitting parameters for the smartphone-acquired tracheal sounds' amplitude and airflow are presented in Table 1 for each respiratory phase and the two models of smartphones. The mean values of the exponent *α* were between *α* = 1 and *α* = 3 for both smartphones. It is worth mentioning that different values of the exponent have been found in different studies, ranging from *α* = 1 [7], *α* = 2 [9], *α* = 3 [8], and values in between this range [2,6]. No statistically significant differences were found between the power law parameters obtained from the Galaxy S4 and the iPhone 4s smartphones with a two-tailed paired t-test with *p* < 0.05 considered as statistically significant (SPSS Statistics 17, IBM Corporation, Armonk, NY, USA).

### 3.2. Breath-Phase Onset Detection Using Tracheal Sounds

**Figure 6.**
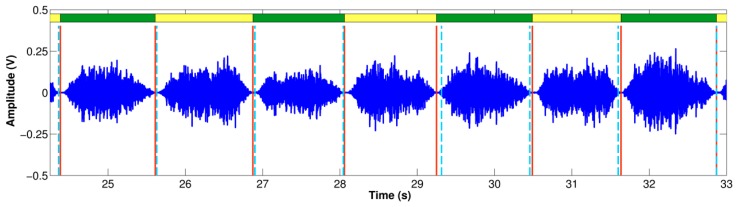
Example of breath-phase onset detection using the tracheal sounds acquired using a smartphone. Solid red lines indicate the breath-phase onsets detected using the volume signal from the spirometer. Dashed blue lines indicate the breath-phase onsets detected using only the information from the acquired tracheal sound.

**Figure 7.**
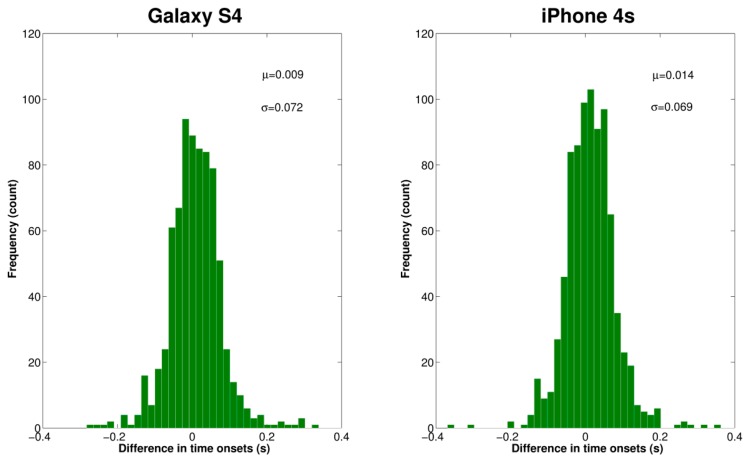
Distribution of the time differences of breath-phase onsets (*Δ_onset_*) detected using the volume signal from the spirometer and breath-phase onsets detected using tracheal sounds acquired with the smartphones.

Table 2 summarizes the results obtained for the breath-onset detection using each smartphone for all volunteers. The absolute time difference between the reference breath-phase onsets from the volume via the spirometer and the estimated breath-phase onsets from the SE of the acquired tracheal sound, |*Δ_onset_*|, was computed. In addition, the total number of true breath-phase onsets computed from the volume is presented together with the corresponding extra and missed breath-phase onsets computed from the tracheal sounds. Note that the total number of true onsets is not the same for both smartphones given that different maneuver trials were performed for each subject. An example of the breathing onset using the iPhone 4s smartphone is shown in Figure 6. As shown, the *Δ_onset_* is not consistently positive or negative. The distribution of time onsets was computed via the histogram and is shown in Figure 7 for each smartphone. We found that on average the breath-phase onsets |Δ_onset_| detected with the smartphones have a time difference of approximately 50 ms from the onsets detected from the volume. Since some onsets were detected before or after the reference onsets, overall these *Δ_onset_* values compensate and the mean onsets differ 9 ms and 14 ms for Samsung S4 and iPhone 4s, respectively. A two-tailed two-sample t-test was performed for *Δ_onset_* and |*Δ_onset_*| obtained from both smartphones for the total number of onsets. No statistically significant differences (*p* > 0.05) were found between *Δ_onset_* and |*Δ_onset_*| computed from the Galaxy S4 and iPhone 4s.

### 3.3. Instantaneous Respiratory Rate (IRR) Estimation Using Tracheal Sounds

**Figure 8.**
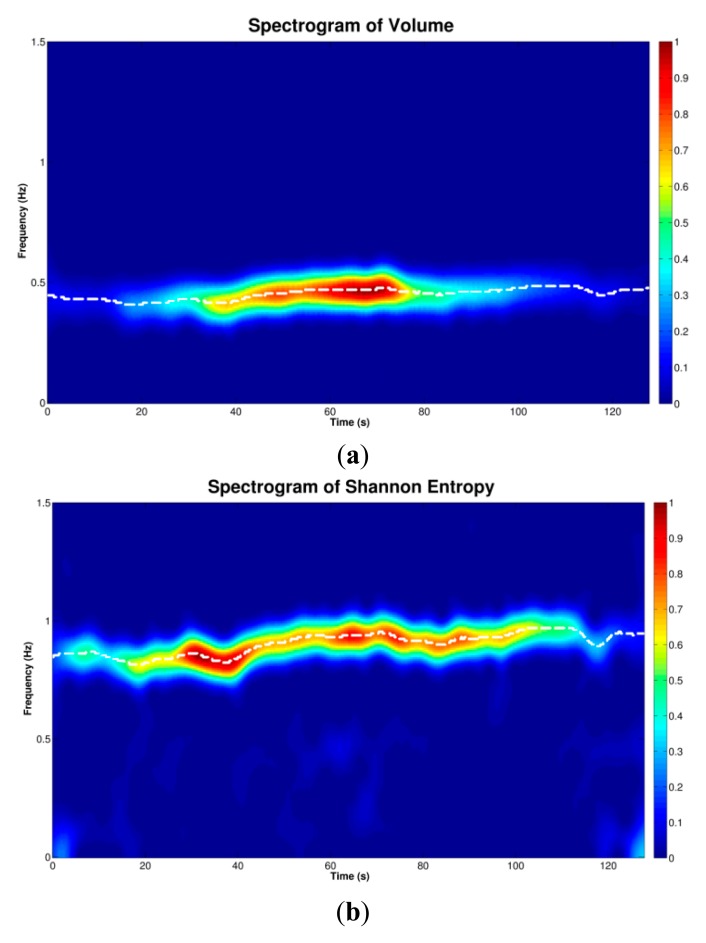

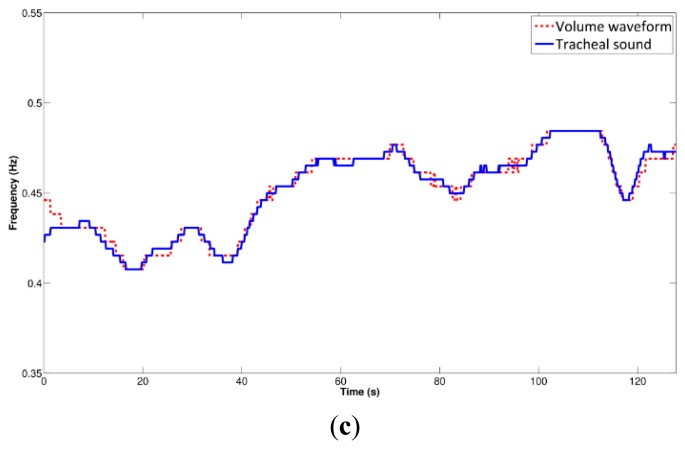
Estimation of the instantaneous respiratory rate using tracheal sounds acquired with a smartphone. (**a**) Spectrogram of the volume signal from the spirometer; (**b**) Spectrogram of the Shannon entropy of tracheal sound acquired with a smartphone. Observe that the main frequency content of the entropy signal is located at twice the frequency of that from the volume signal. White dashed lines indicate the maximum peak at each time instant; (**c**) Instantaneous respiratory rate computed from corresponding spectrograms of volume and Shannon entropy of tracheal sound.

**Figure 9.**
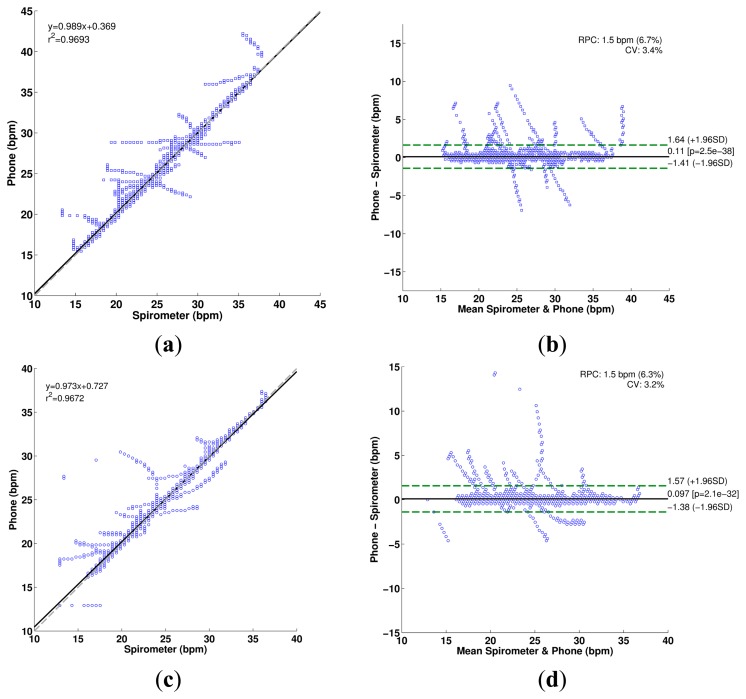
Comparison of instantaneous respiratory rate estimated from tracheal sounds acquired with smartphones and estimated from volume signals (*N*=9 subjects). (**a**) Regression line for estimation from Galaxy S4; (**b**) Bland-Altman plot for estimation from Galaxy S4; (**c**) Regression line for estimation from iPhone 4s; (**d**) Bland-Altman plot for estimation from iPhone 4s. In regression plots, the grey dashed line indicates the identity line and the solid black the regression line. In Bland-Altman plots, the solid black line indicates the bias while the dashed green lines indicate the 95% limits of agreement.

The IRR estimation process using the spectrogram is illustrated in Figure 8 for a tracheal sound acquired using the iPhone 4s. It can be seen that the main frequency of the SE of the tracheal sound (Figure 8b), is located at twice the main frequency of the volume (Figure 8a), which is considered as reference, as the SE resembles a rectified airflow signal. At each time instant, the frequency at which the maximum energy of the TFR occurs was extracted from the corresponding spectrogram (white dashed lines superimposed on TFRs). Comparison of the estimated instantaneous frequencies of the SE of tracheal sound and volume of the spirometer is shown in Figure 8c. In most cases, we found that the discrepancies were more notable at the beginning and the end of the signal where the airflow levels were lower which in turn provided tracheal sound signals with small amplitudes. These are reflected as dispersion points in Figure 9. Table 3 summarizes the IRR results for all the subjects in terms of the performance indices for each smartphone. For both smartphones we found high cross correlation coefficients between the IRR estimated from tracheal sounds and volume. This is also reflected in the regression lines in Figure 9a,c for the Galaxy S4 (*r^2^* = 0.9693) and iPhone 4s (*r^2^* = 0.9672), respectively. High linear correlation has been also found between a tracheal acoustical method and pneumotachometer (*r^2^* = 0.98) [41]. A two-tailed paired t-test was performed for each performance index obtained from both smartphones for all subjects. For all performance indices, no statistically significant differences (*p* > 0.05) were found between the results from Galaxy S4 and iPhone 4s for the estimation of IRR considering the volume from spirometer as reference. The regression lines and the Bland-Altman plots between the estimated instantaneous respiration rate from tracheal sounds and the reference instantaneous respiration rate from volume signals are presented in Figure 9 for the Galaxy S4 and iPhone 4s smartphones. Compared to the spirometer, the bias ± 1.96SD and 95% limits of agreement were 0.11 ± 1.52 bpm and −1.41 to 1.63 bpm for the Galaxy S4, and 0.097 ± 1.47 bpm and −1.38 to 1.57 bpm for the iPhone 4s. Similar correlations and limits of agreement have been reported for a commercial device in post-anesthesia patients in comparison to capnography [42].

## 4. Conclusions

In this paper, we propose the use of smartphones to develop a CORSA system that satisfies the current standards in the field. In particular, we employed two market-leading smartphones, the Galaxy S4 and iPhone 4s, and specifically-designed respiratory acoustical sensors for the acquisition of tracheal sounds. We obtained tracheal sounds from healthy volunteers in airflow controlled conditions from 0.5 to 2.5 L/s in a quiet room, but not an anechoic chamber.

The relationship between amplitude of tracheal sounds and airflow has been shown to be useful for respiratory health monitoring [30,31,43]. Tracheal sounds have been used for estimation of the airflow and volume in a non-invasive way [30,31,43]. The tracheal sounds have been used to estimate ventilation parameters by first estimating the airflow and then integrating this signal to estimate the volume [43]. Several features have been used to estimate the airflow from the tracheal acoustical information, and the Shannon entropy of the tracheal sounds was found to provide better performance compared to other models based on the signal envelope and average power [30,31]. In this work, we found that smartphone-acquired tracheal sounds' amplitude is proportional to the airflow from a spirometer in a power law relationship which is in agreement to prior studies [2,6–9]. The power law models found for the inspiratory phase were *A* = (0.450 ± 0.218)*F*^(2.380 ± 1.077)^ for the Galaxy S4, and *A* = (0.371 ± 0.197)*F*^(2.686 ± 0.959)^ for the iPhone 4s, while for the expiratory phase they were *A* = (0.523 ± 0.181)*F*^(1.939 ± 0.900)^ for the Galaxy S4, and *A* = (0.349 ± 0.162)*F*^(2.632 ± 0.711)^ for the iPhone 4s.

Apnea monitoring and automatic breath-phase detection have been other applications of tracheal sounds analysis [44,45]. In particular, information from the logarithm of the variance of the tracheal sounds was used as a way to detect the breath-phase onset which becomes a crucial part in an automatic acoustical system. Towards this goal, we tested the ability of the smartphone-acquired tracheal sounds to detect the breath-phase onsets, as this processing stage is important when the acoustical approach is used for airflow measurement and automatic breath-phase classification. Our results indicate that on average the onsets estimated from the smartphone-acquired tracheal sounds differ by only 52 ± 51 ms for Galaxy S4, and 51 ± 48 ms for iPhone 4s, from the corresponding onsets detected from the spirometer reference signal.

Estimation of the respiratory rate using an acoustical approach has recently gained popularity in clinical settings. As a vital sign, respiration rate can be used to predict serious clinical events [46]. In particular, continuous monitoring of breathing status becomes relevant to identify and predict risk situations both inside and outside clinical settings. Current clinical continuous monitoring methods include qualified human observation, impedance pneumography, and capnography monitoring. However, these methods have disadvantages, *e.g*., low tolerance of the patient for using the nasal cannula, or leaks around this cannula in capnography. As an alternative, respiratory rate estimation based on tracheal sound has been proposed [41]. Recently, a commercial device that monitors the respiratory rate via tracheal sounds was introduced for clinical settings (Masimo Rainbow SET^®^ Acoustic Monitoring, Masimo Corp., Irvine, CA, USA). The accuracy of this device has been tested against capnography and good correlation has been found between both methods [42,47]. However, there is still a need for a small and discrete device for everyday use, able to estimate the respiration rate in a continuous and non-invasive way outside the clinical setting [48]. Towards addressing this need, we found good correlation between the smartphone-based respiratory rate estimates and the spirometer-based ones (*r^2^* ≈ 0.97), as well as 95% limits of agreement ranging approximately from −1.4 to 1.6 bpm for subjects breathing in a range from 15 to 35 bpm. Overall we did not find statistically significant differences between the results from the Galaxy S4 and iPhone 4s devices.

By employing smartphone devices we were able to reproduce major findings in the tracheal sounds field obtained with conventional CORSA systems. We foresee that efforts similar to the one performed in this study would result in a reliable, low-cost, and easy-to-upgrade portable system that could aid not only general practitioners but also serve as on-demand health monitors outside clinical settings. In addition, systems with such characteristics would aid in the acquisition of large-sample studies in locations not easily accessible nowadays with the currently-used CORSA systems.

Our future work includes implementation of the presented signal processing techniques into applications on the smartphone operating systems, *i.e*., Android and iOS, which will govern the acquisition, processing and display of the tracheal sounds information.

## Figures and Tables

**Table 1.** Results of the smartphone-acquired tracheal sounds amplitude and airflow relationship using a model of the form *A* = *kF^α^* (*N* = 9 subjects).

Respiratory Phase	Parameter	Galaxy S4	iPhone 4s
*Inspiration*	*k*	0.450 ± 0.218	0.371 ± 0.197
	*α*	2.380 ± 1.077	2.686 ± 0.959
*Expiration*	*k*	0.523 ± 0.181	0.349 ± 0.162
	*α*	1.939 ± 0.900	2.632 ± 0.711

Values presented as mean ± standard deviation.

**Table 2.** Results of the breath-phase onset detection using smartphone-acquired tracheal sounds in comparison to those detected from volume signal (*N*= 9 subjects).

Parameter		Galaxy S4	iPhone 4s
|*Δ_onset_*|	[*s*]	0.052 ± 0.051	0.051 ± 0.048
*Total onsets*		767	854
*Extra onsets*		12	5
*Missed onsets*		5	6

Values presented as mean ± standard deviation.

**Table 3.** Results of the instantaneous respiratory rate estimation using tracheal sounds acquired with the smartphones in comparison to those from volume signals (*N* = 9 subjects).

Parameter		Galaxy S4	iPhone 4s
*ρ*	[*unitless*]	0.9994 ± 0.0004	0.9995 ± 0.0004
*RMSE*	[*bpm*]	0.731 ± 0.2878	0.700 ± 0.367
*NRMSE*	[*%*]	3.218 ± 1.297	2.957 ± 1.322

Values presented as mean ± standard deviation.
